# Challenges and Opportunities for Precision Surgery for Colorectal Liver Metastases

**DOI:** 10.3390/cancers16132379

**Published:** 2024-06-28

**Authors:** Robert Michael O’Connell, Emir Hoti

**Affiliations:** Department of Hepatopancreaticobiliary and Transplantation Surgery, Saint Vincent’s University Hospital, D04 T6F4 Dublin, Ireland

**Keywords:** liver resection, colorectal liver metastases, precision surgery

## Abstract

**Simple Summary:**

Colorectal cancer is a common illness. It can spread to the liver in about a quarter of people with colorectal cancer, known as metastases. Previously, people with liver metastases did not survive for long. Thankfully, this is changing. As we understand more about the underlying causes and genetics of the disease, we can tailor treatments to patients on a more individual basis. Treatments like chemotherapy have made a difference in patients’ survival, and now newer treatments like immunotherapy can have even greater benefits. Surgery is also changing, with more advanced techniques allowing for better recovery for patients and more aggressive surgery. It is important for surgeons to consider a large number of individual factors when making decisions with patients about their treatment—this is what we mean by “precision surgery”.

**Abstract:**

The incidence of colorectal cancer and colorectal liver metastases (CRLM) is increasing globally due to an interaction of environmental and genetic factors. A minority of patients with CRLM have surgically resectable disease, but for those who have resection as part of multimodal therapy for their disease, long-term survival has been shown. Precision surgery—the idea of careful patient selection and targeting of surgical intervention, such that treatments shown to be proven to benefit on a population level are the optimal treatment for each individual patient—is the new paradigm of care. Key to this is the understanding of tumour molecular biology and clinically relevant mutations, such as KRAS, BRAF, and microsatellite instability (MSI), which can predict poorer overall outcomes and a poorer response to systemic therapy. The emergence of immunotherapy and hepatic artery infusion (HAI) pumps show potential to convert previously unresectable disease to resectable disease, in addition to established systemic and locoregional therapies, but the surgeon must be wary of poor-quality livers and the spectre of post-hepatectomy liver failure (PHLF). Volume modulation, a cornerstone of hepatic surgery for a generation, has been given a shot in the arm with the advent of liver venous depletion (LVD) ensuring significantly more hypertrophy of the future liver remnant (FLR). The optimal timing of liver resection for those patients with synchronous disease is yet to be truly established, but evidence would suggest that those patients requiring complex colorectal surgery and major liver resection are best served with a staged approach. In the operating room, parenchyma-preserving minimally invasive surgery (MIS) can dramatically reduce the surgical insult to the patient and lead to better perioperative outcomes, with quicker return to function.

## 1. Introduction

Colorectal cancer is among the most common malignancies globally, with over 1.9 million new diagnoses and 930,000 deaths in 2020 alone [1]. The annual incidence is expected to increase to 3.2 million globally by 2040 [2]. There is a clear correlation between a higher human development index and the incidence of colorectal cancer [3]. While increasing age has long been a risk factor for the development of colorectal cancer, the incidence of colorectal cancer is increasing in those under the age of 50 years, particularly in high-income countries [4]. Indeed, by 2030, the incidence rate for colon and rectal cancer is expected to increase by 90.0% and 124.2% for patients 20 to 34 years of age and by 27.7% and 46.0% for patients 35 to 49 years of age, respectively [5].

Environmental risk factors play a key role in the increasing incidence of colorectal cancer among younger people in high-income countries. Approximately 65% of all colorectal cancer is sporadic and occurs in the absence of germline genetic causes or significant family history due to the impact of environmental causes on tumourigenesis [6]. The prevalence of obesity is one such risk factor, and increasing obesity among younger adults is associated with a higher risk of colon cancer [7]. The so-called “Western diet”—a diet with added sugar, refined grains, and a relatively higher intake of red and processed meat—is a significant risk factor for developing colorectal cancer, with a relative risk (RR) of 1.31 (95% CI, 1.15–1.48) when compared to a more prudent diet rich in fruit, vegetables, fish, poultry, and wholegrain products [8]. Cigarette smoking is a risk factor for colorectal cancer, and smokers with colorectal cancer have been shown to have a higher risk of mortality (HR = 1.11, 95% CI = 1.05–1.19) [9]. Alcohol consumption is associated with an increasing risk of developing colorectal cancer in a dose-dependent manner, with heavy drinkers (those consuming >50 g/day of ethanol) most at risk [10].

A total of 20–30% of cases of colorectal cancer are considered to be familial, whereby affected people have a significant family history of colorectal cancer without a defined hereditary syndrome [11]. Those people with at least one affected first-degree relative have an approximately twofold increased risk of developing colorectal cancer when compared to the general population (RR2.05 (95% CI 1.96–2.14)) [12]. On the other hand, 3–5% of colorectal cancer cases are related to inherited syndromes such as hereditary nonpolyposis colorectal cancer syndrome (also known as Lynch syndrome), familial adenomatous polyposis (FAP) syndrome, or MUTYH-associated polyposis [13].

Approximately 25% of patients with colorectal cancer, overall, will develop colorectal liver metastases (CRLM), with approximately 15% of patients having synchronous CRLM at presentation and 19% developing metachronous CRLM [14,15,16]. Only approximately 20% of patients with CRLM have resectable disease; however, this proportion is increasing with modern systemic therapy, improvements in volume optimisation, and advanced surgical techniques [17]. The five-year overall survival rate post-resection for CRLM is typically reported to be around 50%, while a 10-year survival rate of 30% is reported [18,19]. Multimodal and multidisciplinary treatment, with surgery as a cornerstone, has been shown to provide an effective cure for 20% of people with resectable CRLM [20].

In an era of an increased understanding of tumour biology and individual patient risk factors, expanding systemic and targeted treatment options, and improving surgical technology, surgical oncologists have the opportunity to offer “precision surgery” to their patients with CRLM. Precision surgery was defined by Jones et al. as not only absolute adherence to tissue planes, the recognition of critical anatomy, and a fastidious technique but also careful patient selection and the targeting of surgical interventions to ensure that interventions that are proven to provide benefit on a population level are the optimal treatment for each individual patient [21].

## 2. Patient Selection and Defining Resectability

Historic criteria for defining the resectability of CLRM were primarily anatomical. In order to be considered resectable, the surgeon must be able to achieve a clear margin (R0), spare two adjacent segments, preserve vascular inflow, outflow, and biliary drainage, and leave sufficient liver volume (>20% future liver remnant (FLR) in otherwise healthy patients [22]. Interestingly, despite these relatively clear criteria, there is significant inter-surgeon variability in the assessment of resectability [23]. As criteria for resectability encompass more factors, most particularly patient and tumour biology, the role of a multidisciplinary team becomes more critical to preventing the undertreatment of those patients with potentially resectable disease [24].

### 2.1. Clinical Scoring Systems

The inadequacy of anatomical criteria alone to select patients with CRLM for resection led to a number of prognostic scoring systems being developed in the 1990s, most notably the clinical risk score (CRS) developed by Fong et al. [25,26]. The authors defined the following five factors associated with poorer long-term survival: a node-positive primary tumour; a disease-free interval from primary to metastasis diagnosis of <12 months; a number of tumours > 1; a largest hepatic tumour diametre of >5 cm; and carcinoembryonic antigen (CEA) > 200 ng/mL. Each factor present was given a score of one, and higher scores were associated with worsening long-term outcomes. In an era before the availability of molecular biological markers, the scoring system was used as a surrogate marker for tumour biology [27]. Ultimately, the CRS, along with other prognostic scoring systems based on clinical factors, has limitations in predicting long-term prognosis [28,29].

### 2.2. Tumour Molecular Biology

The availability of next-generation sequencing (NGS) allows for the molecular profiling of individual patient tumours to identify molecular biomarkers with significant treatment and prognostic value in colorectal cancer [30]. A number of different mutations can be seen in colorectal cancer, such as mutations of RAS, BRAF, PIK3CA, TP53, APC, B-Catenin, and SMAD 4 [31].

RAS proteins are proto-oncogenes that regulate cell proliferation and cell survival and are encoded by the following three genes: KRAS, NRAS, and HRAS [32]. KRAS mutations are identified in approximately 30% of people with CRLM [33]. Overall, for patients with colorectal cancer, the impact of KRAS mutations on survival is not fully clear—certainly, those with KRAS mutations on codon 12 have worse outcomes, but those with codon 13 mutations may have similar outcomes to patients with wild-type KRAS [34]. For patients undergoing resection for CRLM, a KRAS mutation is negatively associated with OS (HR 1.674; 95% CI 1.341–2.089; p < 0.001) and is also negatively associated with RFS (HR 1.529; 95% CI 1.287–1.817; p < 0.001) [35]. Margonis et al. showed that G12V (HR 1.78, 95% CI 1.00–3.17; p = 0.05) and G12S (HR, 3.33; 95% CI, 1.22–9.10; p = 0.02) mutations on codon 12 are specifically associated with worse outcomes following resection for CRLM than wild-type KRAS [36]. The presence of an RAS mutation may require tailoring of the operative approach to the patient, and additional consideration must be given when considering anticipated margin or ablative therapies, as discussed below [37].

BRAF mutations are seen in about 10% of patients with metastatic colorectal cancer and typically occur together with RAS mutations [38]. The V600E mutation of BRAF has been shown to have significantly worse outcomes for patients with CRLM undergoing resection with OS (hazard ratio [HR], 2.76; 95% CI, 1.74–4.37; p < 0.001) and DFS (HR, 2.04; 95% CI, 1.30–3.20; p = 0.002) [39]. BRAF-mutated tumours tend to be aggressive and poorly responsive to chemotherapy, but despite this, a resection of CRLM can convey a survival advantage [40].

APC mutation is characteristically seen in FAP but also occurs in 50% of patients with CRLM [27]. APC mutations do not seem to influence survival on their own; however, when combined with PIK3CA, they confer significantly worse overall survival (HR 3.09; p < 0.001) for patients undergoing CRLM resection [41].

TP53 mutations are encountered in over 60% of patients with colorectal cancer [42]. The incidence is higher in those patients with CRLM, with p53 mutations present in 70–80% [43,44]. A deleterious impact of TP53 mutation on survival post-resection for CRLM has not been universally shown in the literature. Bao et al., for example, showed no impact on post-liver resection OS (HR: 1.41 95% CI 0.72–2.75 p = 0.313) [43]. However, co-existent TP53 and RAS mutations are independent predictors of poor overall survival (HR 2.62 95% CI 1.41–4.87 p = 0.002), particularly when the RAS mutation is co-existent with a high-risk EAp53 mutation (5-year OS 12.2% vs. 55.7% for TP53wt, p < 0.001) [45].

SMAD 4 mutations occur in up to 20% of patients with colorectal cancer and are associated with poorer OS and an increased likelihood of co-existent RAS mutations [46]. Approximately 15% of patients with CRLM will have SMAD 4 mutations, which are associated with poorer OS and DFS [47]. The presence of SMAD 4 mutation is associated with a worse 3-year OS post-resection of CRLM compared to SMAD 4wt (62% vs. 82%, p < 0.0001) and in those with CRLM undergoing systemic therapy alone (22% vs. 38% p = 0.012) [48].

A further molecular marker of some importance for CRLM is microsatellite instability (MSI). Deficiencies with DNA mismatch repair (MMR) proteins, such as MLH1, PMS2, MSH2, and MSH6, cause errors during DNA replication, which in turn, give rise to MSI [49]. MSI occurs in about 15% of colorectal cancers, and of those, 20% are associated with Lynch syndrome [50]. MSI is associated with poorer OS (HR: 1.21 95% CI: 1.01–1.46, p = 0.04) for patients following resection for CRLM compared to MSS [51].

Molecular biomarkers have been incorporated into a number of updated prognostic scoring systems, potentially replacing the CRS. Brudvik et al. developed a modified clinical score (m-CS) that incorporated RAS mutation status, primary tumour nodal positivity, and largest liver lesion ≥50 mm and outperformed the CRS in predicting survival after resection for CRLM [52]. Margonis et al. developed the genetic and morphological evaluation (GAME) score, which allocated scoring as follows: KRAS mutated tumours: 1 point; carcinoembryonic antigen (CEA) ≥20 ng/mL: 1 point; primary tumour lymph node metastasis: 1 point; ≤ tumour burden score (TBS) < 9: 1 point or TBS ≥ 9: 2 points; and extrahepatic disease: 2 points [53]. This incorporates the TBS, which accounts for the cumulative impact of tumour size and tumour number on survival and is defined as TBS^2^ = (diametre of the largest lesion)^2^ + (number of liver lesions)^2^ [54]. Wong et al. compared GAME, CRS, and m-CS in a single-centre retrospective cohort study and showed that GAME was the best-performing score for predicting OS [55]. External validation of the GAME score, compared with the CRS, showed that the discriminative ability for the OS of CRS was 0.577 (95% CI [0.554, 0.601]), and for GAME, it was 0.596 (95% CI [0.572, 0.621]), with both showing insufficient preoperative risk stratification for clinical decision-making [56].

At present, traditional selection criteria, along with an improved understanding of molecular biomarkers, do not provide sufficient data to select patients for resection for CRLM, and there are other factors that the surgeon must consider, most notably, the fitness of the patient and the behaviour of the tumour. Despite this, understanding tumour molecular biology is fundamental for the modern surgeon oncologist and should be considered part of the gold standard for preoperative assessment in the era of precision surgery [57].

### 2.3. Patient Fitness

As discussed above, CRLM is a disease most commonly seen in older adults and in those with obesity. Selecting suitably fit patients for surgery is, therefore, a key step in offering precision surgery for CRLM. An American Society of Anesthesiologists (ASA) score ≥3 has been shown to increase the risk of major perioperative morbidity for patients undergoing liver resection (OR 1.4, p = 0.001) [58]. In addition, data from the Surveillance, Epidemiology and End Results (SEER) database in the United States show that patients with a Charlson comorbidity index score ≥1 have marginally worse long-term outcomes following hepatectomy for CRLM (HR 1.07, 95% CI 1.06–1.08, p < 0.001) [59].

Older age is in itself not a contraindication to liver resection, and CRLM in older adults may have different, more indolent behaviour than in younger people [60]. Older patients with CRLM can benefit from improved OS and DFS with liver resection [61]. However, postoperative morbidity is higher in patients aged >75 years (21 vs. 32%; p = 0.001), and postoperative mortality is higher in both patients aged >70 years (2 vs. 4%; p = 0.01) and in patients aged >75 years (1 vs. 6%; p = 0.02), compared to younger cohorts [62].

Frailty is a more important prognostic marker than age alone and can be associated with a significantly increased risk of perioperative morbidity [63]. Osei-Bordom et al. showed that patients with frailty had significantly higher 90-day mortality (6.6% vs. 2.9%, p < 0.001) and postoperative complications (36.3% vs. 26.1%, p < 0.001) following liver resection [64]. Frailty can also impact long-term outcomes, with frail patients showing significantly worse 3-year survival post-resection for CRLM than patients without frailty (69.3% vs. 91%, p < 0.01) [65].

While obesity is a risk factor for the development of colorectal cancer, there is no difference in perioperative or long-term outcomes between obese and non-obese patients undergoing hepatectomy for CRLM [66]. Fischer et al. showed that diabetes was an independent predictor of morbidity (OR = 2.44, p = 0.02), Clavien–Dindo grade IV complications (OR = 3.6, p = 0.004), unplanned readmission (OR = 2.44, p = 0.04), and bile leakage (OR = 2.06, p = 0.046) for patients undergoing major hepatectomy [67].

Sarcopenia is increasingly recognised as a risk factor for worse perioperative outcomes following hepatectomy for CRLM [68]. A crucial aspect in the definition of sarcopenia is that it encompasses not only a loss of muscle mass but also of muscle strength and function [69]. Patients with sarcopenia are at greater risk of chemotherapy toxicity and experience associated reductions in treatment efficacy due to interruptions and dose reductions [70]. Chemotherapy is associated with a median muscle area loss of 6% in patients with metastatic colorectal cancer, and those with >9% muscle area loss have a significantly worse OS [71]. Additionally, a metanalysis by Waalboer et al. showed that both low muscle mass (hazard ratio (HR) 1.35, 95% CI 1.08–1.68) and low muscle density (HR 1.97, 95% CI 1.07–3.62) were associated with impaired OS post liver resection for CRLM [72].

### 2.4. Response to Systemic Therapy

The timing of chemotherapy administration in the management of CRLM is still a subject of debate. However, societal guidelines, such as the recently published European consensus guidelines, suggest that systemic chemotherapy, and not surgery, is the preferred first treatment for synchronous disease [73]. Certainly, upfront chemotherapy confers the advantage of allowing an assessment of the tumour biology, and a lack of response to, or progression on, chemotherapy suggests disease with an overall poor prognosis, potentially allowing patients to avoid futile liver resection [74].

The EPOC trial was the first major randomised trial examining perioperative chemotherapy for resectable CRLM, in which 364 patients were randomised to either receive six cycles of FOLFOX4 pre- and post-operatively or surgery alone for CRLM, with the primary outcome being PFS [75]. The 3-year PFS was 35.4% in the perioperative chemotherapy group, compared to 28.1% in the surgery alone group (p = 0.058), while it was 42.4% versus 33.2% (p = 0.025), respectively, when only patients who underwent resection were included (152 per group). Subsequent long-term follow-up revealed no difference in five-year survival between the groups (51.2% vs. 47.8%, p = 0.34); however, there was a sustained difference in PFS when ineligible patients were excluded (HR 0.78, 95% CI 0.61–0.99, p = 0.035) [76]. The subsequent NewEPOC trial, in which cetuximab plus standard perioperative chemotherapy was compared to standard perioperative chemotherapy in patients without KRAS mutations, was terminated early because of significantly worse PFS in the cetuximab plus chemotherapy group (14.1 months (95% CI 11.8–15.9) vs. 20.5 months (95% CI 16.8–26.7), HR1.48, 95% CI 1.04–2.12, p = 0.030) [77]. Subsequently published long-term follow-up data from the NewEPOC trial show significantly worse OS in the cetuximab plus chemotherapy group compared to chemotherapy alone (HR 1.45, 95% CI 1.02–2.05; p = 0.036) [78].

Assessing tumour radiographic response to chemotherapy is typically performed using the response evaluation criteria in solid tumours (RECIST) criteria, but for CRLM, modified criteria (mRECIST) based on morphology rather than size alone is the standard tool [79]. Radiological response to treatment has been shown to be a significant prognostic factor. Gruenberger et al. evaluated outcomes for 50 patients after six cycles of preoperative XELOX or FOLFOX4 and showed that median RFS was significantly influenced by tumour response, with 24.7 months (95% CI: 4.50 to 44.97) in responding patients, 8.2 months (95% CI: 3.09 to 13.31) in patients with stable disease, and 3.0 months (95% CI: 0 to 8.91) in patients with progressive disease (p < 0.004) [80]. These findings have been corroborated by other studies; Ruzzenente et al. showed that for patients with a TBS ≥ 6, response to preoperative chemotherapy (defined as a decrease in the TBS by 10% or more) was associated with a significantly improved 5-year OS of 48.9% (95% CI, 33.3–71.8) compared to 26.6% (95% CI, 19.7–35.9) for those without a response (p = 0.021) [81]. Vigano et al. showed that up to 17% of patients with a disease that is stable or responsive to chemotherapy can progress in the short interval between completing preoperative therapy and undergoing hepatectomy, and these patients face poor outcomes, with a 3-year overall survival (OS) of 23.0 vs. 52.4% (p < 0.001) [82]. Brunsell et al. showed that the 5-year OS for patients with progression of disease on chemotherapy was significantly worse compared to patients with partial response, but also that patients with “heterogenous response” (a patient with disease progression in some lesions while radiographic response in others) have significantly worse survival than those with global response (median survival 23 vs. 50 months, p = 0.003) [83].

Elevated CEA levels have long been recognised as a prognostic factor for resectable CRLM, indeed, the original CRS included CEA as a variable [25]. Elevated preoperative CEA levels have been shown to be associated with worse PFS and OS [84,85]. Additionally, the responsiveness of CEA levels to chemotherapy has been shown to be a predictor of survival for patients with metastatic unresectable CRLM [86]. De Haas et al. showed that CEA response closely predicts radiological response to preoperative chemotherapy but did not significantly predict long-term survival in their cohort, while an increase in Ca 19.9 levels of >20% during preoperative chemotherapy was predictive of worse OS [87]. Stremitzer et al., on the other hand, showed that a decrease in a previously elevated CEA by >50% during neoadjuvant therapy was a significant predictor of post-hepatectomy OS (HR 0.37, p = 0.025) [88].

Circulating tumour DNA (ctDNA) is an emerging biomarker in CRLM and has been shown to predict postoperative recurrence following resection for CRLM [89]. Wehrle et al. showed that the absence of detectable ctDNA post-resection for CRLM is associated with a dramatically lower risk of short interval recurrence than for those patients with detectable ctDNA (0% vs. 57%, p = 0.419) [90]. Wang et al. showed that early ctDNA response to neoadjuvant therapy is a significant predictor of overall treatment response and outcomes, outperforming traditional tumour markers [91]. The use of ctDNA response to neoadjuvant chemotherapy to select patients for resection of CRLM is still an emerging concept, but poor ctDNA response to preoperative therapy may be able to identify those patients unlikely to benefit from hepatectomy and so avoid unnecessary surgery [92].

## 3. The Emergence of Immunotherapy and Its Impact on Resectability

Interruption of the regulatory T cell-mediated immune response is one of the main immune evasion techniques used by cancer cells, and the recent emergence of immunotherapy to combat this represents a significant development in the management of metastatic colorectal cancer [93]. Pembrolizumab and nivolumab are two monoclonal antibodies that block the PD-1 immunosuppressive pathway, and along with ipilimumab, which blocks the similarly inhibitory CTLA-4 pathway, have shown significant potential in treating metastatic colorectal cancer [94]. These therapies are also referred to as immune checkpoint inhibitors and counteract the attenuated T cell immunologic response seen in malignancy [95].

MMR status is an important predictor of response to immunotherapy for patients with metastatic colorectal cancer. Le et al., in a phase II trial, showed that patients with MMR-deficient metastatic colorectal cancer had significantly improved survival with pembrolizumab than those with MMR-intact tumours (HR of death 0.22, p = 0.05) [96]. The phase II CheckMate 142 study examined nivolumab and low-dose pembrolizumab as a combined regimen in patients with MSI-high/MMR-deficient metastatic colorectal cancer and showed a disease control rate of 84% (95% CI, 70.5 to 93.5) [97]. The KEYNOTE-177 phase III RCT compared first-line pembrolizumab against 5-FU-based chemotherapy with or without bevacizumab and cetuximab in patients with MSI-high/MMR-deficient metastatic colorectal cancer, which showed significantly improved PFS on pembrolizumab in the interim analysis (HR 0.60; 95% CI, 0.45 to 0.80, p = 0.0002) [98]. After a median of 44.5 months, the pembrolizumab arm showed improved OS (HR 0.74, 95% CI 0.53–1.03, p = 0.036), but this failed to meet the pre-specified α of 0.025 to show statistical significance [99].

Immunotherapy has shown exciting potential in the neoadjuvant setting, with single-agent dostarlimab, an anti–PD-1 monoclonal antibody, associated with complete clinical response in 12/12 patients with locally advanced MMR-deficient rectal cancer in a phase II trial [100]. The NICHE study examined the role of the neoadjuvant combination of ipilimumab and nivolumab in early colorectal cancer, showing a pathological response in 20/20 patients with MMR-deficient disease, including 12 complete pathological responses, while only 4/15 patients with MMR-intact disease had a pathological response [101]. Trial data on the role of preoperative immunotherapy for CRLM are scant, however. Kanikarla Marie et al. describe a pilot trial using a combination of neoadjuvant anti-CTLA-4 and anti–PD-1 therapy for 24 patients with CRLM, with a complete response in four patients and an OS of 24.5 months (95% CI: 16.5–28.4) [102]. To date, most evidence supporting the use of neoadjuvant immunotherapy in CRLM comes from limited case series, in which it has shown a role in converting previously unresectable disease to resectable disease [103]. Larger studies, and randomised evidence, will be needed to fully establish the role of immunotherapy in the treatment paradigm for CRLM, but it represents an opportunity for precision individualised treatment going forward [104].

## 4. Hepatic Artery Infusion and Converting CRLM to Resectable Disease

Hepatic artery infusion (HAI) allows for the delivery of chemotherapeutic agents to the liver, utilising the hepatic metabolism to produce minimal systemic toxicity [105]. For unresectable disease, HAI with systemic therapy has been shown to have comparable long-term outcomes to multiagent chemotherapy regimens such as FOLFOXIRI, but with a favourable toxicity profile [106]. It is important to note that HAI should be combined with some systemic treatment to prevent the progression of extrahepatic disease [107].

While HAI clearly has a role in palliation, its role in converting unresectable CRLM to resectable disease is an emerging paradigm. Kemeny et al. showed that HAI with floxuridine/dexamethasone, combined with systemic oxaliplatin and irinotecan, converted 47% of patients with previously unresectable CRLM to resection [108]. Conversion to resection was associated with a significantly improved OS HR0.16 (95% CI 0.05–0.49, p = 0.001) [109]. Longer-term follow-up from the same group shows that 52% of patients converted to resectable disease, with a 5-year OS of 36%, and 51% in chemotherapy-naïve patients [110]. The OPTILIV multicentre phase II trial included patients with wtKRAS and unresectable CRLM who underwent HAI with irinotecan, oxaliplatin, and 5-FU (HAI-FOLFIRINOX), along with systemic cetuximab, with a 40.6% response rate (95% CI 28.6–52.3%) and 29.7% converting to resection (18.5–40.9%) [111]. Retrospective data from six French centres show that for patients with unresectable CRLM treated with either HAI-FOLFIRINOX using the OPTIILIV protocol or HAI-oxaliplatin, the secondary liver resection rate was 35.6% with HAI–Folfirinox and 16.7% with HAI–oxaliplatin (p = 0.007) [112]. While large, randomised studies are lacking, there is increasing use of HAI for conversion for unresectable CRLM, with a focus on providing it as part of first-line therapy to increase the ultimate rates of resection [113].

## 5. Impact of Preoperative Therapy on Hepatic Function

While neoadjuvant therapy may confer benefits in terms of resectability and oncological outcome, the surgeon must be wary of the impact of these therapies on hepatic function. With traditional chemotherapy, the pattern of hepatotoxicity depends on the regimen; irinotecan-based chemotherapy regimens increase the risk of steatohepatitis (RR 3.45, 95% CI 1.12–10.62 p = 0.03), while oxaliplatin-based regimens are associated with an increased risk of hepatic sinusoidal injury (RR 2.22, 95% CI 1.47–3.36, p = 0.0002) [114]. A metanalysis by De Meijer et al. showed that patients with >30% hepatic steatosis have an RR of 2.01 (95% CI 1.66–2.44, p < 0.001) for major perioperative morbidity and an RR of 2.79 (95% CI 1.19–6.51, p = 0.02) for mortality [115]. Similarly, hepatic sinusoidal injury is associated with worse postoperative liver function and morbidity [116]. While chemotherapy-associated liver injury (CALI) can improve over time, Vigano et al. showed that a chemotherapy-free interval of at least 270 days was required to reduce the incidence of sinusoidal injury [117]. Interestingly, the addition of bevacizumab in the neoadjuvant setting has been shown to reduce the risk of CALI [118,119].

CALI occurs in a dose-dependent manner, with ≥9 cycles of neoadjuvant chemotherapy associated with a higher risk of hepatotoxicity, without improving pathological response to therapy [120]. Chen et al. showed that the optimal cutoff was five cycles of oxaliplatin-based neoadjuvant chemotherapy, which was associated with an improved median OS of 59 versus 31.8 months (p = 0.008) compared to those having extended preoperative therapy for oligometastatic CRLM [121].

### Immunotherapy-Induced Hepatotoxicity

As described above, immunotherapy works by enhancing the immune response through a number of immune pathways. This can result in immune-mediated adverse effects, particularly affecting the skin, endocrine system, respiratory system, and gastrointestinal tract [122]. Immunotherapy-associated hepatitis can range in severity from mild derangements of transaminases to fulminant hepatitis, which is the second most commonly reported cause of fatal immunotherapy-related adverse events, after pneumonitis [123]. The incidence of immunotherapy-induced hepatotoxicity was shown in a metanalysis by Fu et al. to be approximately 6% overall and 1.5% for severe toxicity, but the rates reported in the literature vary substantially [124]. For example, a retrospective review by Cho et al. found that in patients receiving immune checkpoint inhibitors, such as nivolumab and pembrolizumab, 64.4% experienced hepatotoxicity overall and 10.8% had severe hepatotoxicity [125].

In the era of immunotherapy, more patients are likely to be converted from unresectable to resectable disease, but potentially with worse-quality livers. Patient selection becomes ever more complex to ensure that a good response does indeed translate to resectable disease and long-term benefit.

## 6. Liver Volume: Assessment, Function, and Modulation

Post-hepatectomy liver failure (PHLF) is a potentially devastating complication of liver resection, with a reported mortality risk as high as 80% [126]. Failure to ensure sufficient future liver remnant (FLR) following hepatectomy can result in PHLF [127]. For those patients with normal livers, an FLR of between 20 and 30% is commonly cited as sufficient volume to avoid PHLF, but for those with compromised hepatic function, a larger FLR may be required, with an FLR of 30% and 40% typically recommended for patients with hepatic steatosis and cirrhosis, respectively [128]. CT is the imaging modality relied upon for volumetry, with a good correlation between estimated future liver remnant using 3D reconstructed CT and actual postoperative future liver remnant [129]. Martel et al. have shown, however, that a clinically significant difference in FLR between estimated and actual (defined as a difference of +/−5%) can occur in 31% of patients [130].

Precision surgery demands a more accurate assessment of not only FLR but also hepatic function. Traditional biochemical liver function tests and clinical grading systems, such as Child’s Pugh score, provide limited information in the absence of cirrhosis for patients with CRLM [131].

Indocyanine green (ICG) dye that binds to albumin and is excreted into the bile without intrahepatic conjugation, typically within 20 min of administration [131]. The preoperative non-invasive measurement of ICG clearance using pulse spectrophotometry has been shown to predict patients at risk of PHLF [132]. Schwarz et al. showed that a plasma disappearance rate (PDR) of ICG of <19.5%/minute and a 15 min retention rate (ICG-R15) of >5.6% were predictive of PHLF [133]. The utilisation of ICG clearance as a preoperative screening tool remains controversial, with estimates of its sensitivity ranging from 25% to 83% and specificity ranging from 66.1% to 93.8% for the prediction of PHLF, suggesting that its interpretation should be within the context of a multimodal assessment [134]. One disadvantage of relying on the ICG clearance as an assessment of liver function is that it does not account for intersegmental inhomogeneity in hepatic function, such as that which occurs in intrahepatic shunting, and the segments that are to be preserved may not function as predicted [131,135] Some novel methods of improving the predictive value of ICG have been proposed, such as the ICG-Krem (ICG clearance of liver remnant) calculation proposed by Kobayashi et al., which calculates ICG clearance as a function of predicted future liver remnant based on CT volumetry [136].

The LiMax is a more recently developed test of hepatic function. It relies on the metabolisation of orally administered C-methacetin by CYP1A2 within the liver, and then a recording of the rate of excretion of its metabolite ^13^CO_2_ from the lungs using a facemask [137]. An excretion rate of >315 µg/kg/h was seen in healthy volunteers in the work by Stockmann et al., who proposed a decision tree based on the LiMax, and adjusting for FLR for those patients with LiMax rates of 140–315 µg/kg/h, while those below 140 µg/kg/h are not offered surgery in their decision tree [138]. In an RCT carried out by the same group, the incorporation of LiMax into the preoperative assessment algorithm identifies patients at higher risk of PHLF and is associated with reduced overall morbidity and LOS [139]. Like ICG clearance, however, LiMax is limited by sensitivity and specificity concerns, and a recent multicentre study showed that a combination of aspartate aminotransferase to platelet ratio (APRI) and albumin–bilirubin grade (ALBI) outperformed LiMax in the prediction of grade B and C PHLF [140].

A number of imaging techniques have also emerged to improve the assessment of hepatic function. Hepatobiliary scintigraphy (HBS) is a group of similar techniques that measure the rate of hepatic uptake of a radio-labelled agent, allowing for the assessment of liver function [141]. DeGraaf et al. combined HBS with CT volumetry and showed that an uptake of ^99m^Tc-mebrofenin <2.69%/min/m^2^ within the future liver remnant was predictive of PHLF [142]. This has been subsequently validated in a multicentre cohort study, which showed that a cutoff of <2.7%/min/m^2^ uptake in the FLR was associated with a 12% incidence of grade B/C PHLF, with a PPV of 21% and NPV of 93% [143]. Serenari et al. showed that a cutoff of <2.79%/min/m^2^ in the future liver remnant had a PPV of 75% and NPV of 100% [144]. HBS has a role in identifying those patients who may benefit from volume modulation via portal vein embolisation (PVE) and can improve patient selection beyond simple volumetry [145].

An area of interest is the potential role of Gd–EOB–DTPA-enhanced MRI as a tool to measure hepatic function [146]. Araki et al. showed that an MRI-predicted poor function of the FLR was associated with grade B/C PHLF (OR 0.983, 95% CI 0.958–0.996, p = 0.013) [147]. Data to date remain sparse, but it is an emerging technique under evaluation for a role in patient selection for hepatectomy [148].

### Preoperative Volume Modulation Strategies

In 1990, Makuuchi et al. described a case series in which preoperative portal vein embolisation of the ipsilateral side resulted in contralateral hepatic hypertrophy and allowed for a safely extended hepatectomy for perihilar cholangiocarcinoma [149]. This ability to manipulate the FLR has transformed the approach to hepatectomy, including for CRLM, and allows for more extensive resections [150]. PVE results in an increase in FLR volume by approximately 29–57% [151,152,153,154]. Interestingly, the rate of growth post-PVE has been shown to be a better predictor of PHLF than the overall amount of hypertrophy [153].

A new evolution of this concept is the emergence of simultaneous PVE and hepatic vein embolisation, known as liver venous depletion (LVD). Le Roy et al. showed increased hypertrophy of the FLR (51.2% (±42) vs. 31.9% (±34), p = 0.018) and increased rate of growth compared to PVE alone [155]. A subsequent metanalysis has shown that LVD increases the rate of resectability compared to just PVE (resection rate: 87% vs. 75%, OR 1.92, 95% CI 1.13–3.25, p = 0.015) [156]. The DRAGON-2 RCT is currently underway, comparing resectability rates and long-term outcomes between LVD and PVE [157].

## 7. Selecting an Operative Approach

The second aspect of precision surgery after appropriate patient selection is selecting the operative approach and tailoring it to each individual patient’s needs. In synchronous CRLM, possibly the first decision the surgeon must make is the timing of hepatectomy in relation to resection of the primary (and indeed systemic therapy, as discussed above).

### 7.1. Primary-First Approach

Resection of the colorectal primary prior to liver resection is the most commonly adopted approach and may still be considered the standard approach [74]. There are, of course, significant differences in the management of colon and rectal cancers, but a discussion on the role of neoadjuvant therapy, potentially including radiotherapy, and the difference in managing colonic primary tumours based on their location is beyond the scope of this review [158,159]. The 2023 European consensus guidelines recommend adopting a “bowel-first” approach in the following two settings: firstly, in a patient with a symptomatic primary tumour and/or imminent intestinal obstruction or perforation, and secondly, as part of a staged approach (bowel first, liver second) to tumour clearance in patients with a synchronous disease treated by systemic chemotherapy [73]. In an analysis of 658 patients with synchronous CRLM in Sweden, no difference was seen in long-term OS, whether a primary-first, liver-first, or simultaneous surgical strategy was employed [160]. Metanalyses have also not shown a difference in long-term outcomes between staged and simultaneous approaches, but patients having simultaneous resection may have shorter in-hospital LOS [161]. The METASYNC RCT randomised patients with resectable CRLM to either a primary-first approach or simultaneous resection and showed a significant difference in a median OS of 3.9 years for the primary-first approach versus 5.9 years in the simultaneous approach (p = 0.07) [162]. However, this trial has a number of limitations, not least the difficulty in recruiting patients (105 patients randomised across ten centres over ten years), heterogeneity in the burden of liver disease and colorectal primary location, and failure to account for tumour mutational status, limiting the generalisability of its findings [163].

### 7.2. Liver-First Approach

The liver-first approach to CRLM has been shown to be a safe alternative to the classic strategy of the primary-first approach [164]. Despite this, it is only adopted as the approach of preference in the minority of patients with synchronous CRLM and is more common in patients with rectal cancer or larger liver metastases [165]. The 2023 European consensus guidelines recommend adopting a liver-first approach in the following three scenarios: firstly, when there are specific liver-related criteria, such as borderline resectability after chemotherapy, secondly, for patients with rectal cancer who have had a response to neoadjuvant therapy in which liver resection can be undertaken in the window between the completion of therapy and resection of the rectal primary, and thirdly, for patients with a rectal primary and a complete clinical response to neoadjuvant therapy [73]. The liver-first approach appears particularly attractive in those patients with rectal primaries, particularly as rectal resection is associated with major postoperative morbidity in 7–14% of patients [166]. Additionally, in the era of organ preservation strategies for rectal cancer, 10–60% of patients with rectal cancer will have a complete clinical response, depending on the neoadjuvant strategy and definition of response, and may be acceptable for a watch-and-wait strategy for the primary tumour [167]. A note of caution should also be sounded here—Maki et al. showed that 35% of patients with synchronous CRLM undergoing a liver-first strategy never proceeded to resection of their primary tumour, particularly in those patients with a RAS/TP53 co-mutation, although OS was not different from those patients undergoing primary-first or synchronous resection [168].

### 7.3. Simultaneous Resection

Simultaneous resection of the primary and CRLM offers the attraction of potentially reducing the cumulative perioperative morbidity, LOS, pain, and healthcare costs without compromising long-term outcomes [74]. This is true for patients undergoing less complex hepatectomy or less extensive colorectal surgery, but the incidence of severe morbidity and mortality has been shown to increase incrementally based on the extent of liver resection and complexity of colectomy [169]. A recently published study by Endo et al. showed that for patients with high TBS (>10), simultaneous resection was associated with significantly higher postoperative complications than staged resection (34.0% vs. 14.8%, p = 0.03), but no difference was seen for patients with lower TBS [170]. The long-term outcomes, however, are reassuring, with a network metanalysis showing no difference in OS among the three approaches [171]. Indeed, as discussed above, the METASYNC study showed superior OS for the simultaneous approach over the primary-first approach, although the results of this should be interpreted with caution [162]. Ultimately, it appears that minor hepatectomy can be undertaken simultaneously safely with colorectal resection, or indeed a less complex colonic resection with major hepatectomy, while more extensive liver disease should be managed in a staged approach [73].

### 7.4. Anatomical or Parenchyma-Preserving Approach?

The emergence of intra-operative ultrasound (IOUS) has allowed surgeons to perform parenchyma-preserving surgery (PPS), enabling the targeted resection of liver lesions, while preserving as much FLR as possible [172]. PPS can reduce the need for major hepatectomy by as much as 80% [173]. This can be achieved without compromising margin status or long-term oncological outcomes [174]. Anatomical liver resection is associated with an increased risk of perioperative morbidity when compared to PPS (RR = 2.28, 95% CI: 1.88–2.77, p < 0.001) but has a lower risk of intrahepatic disease recurrence (RR = 0.90, 95% CI: 0.82–0.98, p = 0.021) [175]. Mise et al. showed that for those patients with intrahepatic recurrence, PPS was associated with longer OS and longer survival post-recurrence than anatomical resection [176]. The adoption of the PPS has been a paradigm shift in the thought process around the management of CRLM, and the benefits are seen in patients who have intrahepatic disease recurrence in that the PPS approach allows for more options for future salvage.

### 7.5. Resection Margin

Traditionally, a resection margin of >1 cm was thought to be necessary for good oncological outcomes for CRLM, but that was challenged by evidence showing no difference in outcomes for margins <1 cm with no tumour involvement [177]. This led to the consensus recommendation from the EGOSLIM in the 2015 group that a margin of >1 mm should be adopted as the standard for an R0 resection [178]. It should be noted, however, that patients who undergo liver resection with an R1 margin tend to have a higher tumour burden than those who have an R0 margin, and tumour biology, as much as margin status, may be the factor predicting poorer outcomes [179]. The inability to achieve an R0 margin should not, in itself, be considered a contraindication to surgery—de Haas et al. showed an equivalent 5-year OS between patients with R1 and R0 resections in their cohort, albeit patients with R1 resections had higher intrahepatic recurrence rates (28% vs. 17%; p = 0.004) [180]. Neoadjuvant chemotherapy may protect against any negative impact of an R1 resection; Laurent et al. showed that OS and DFS were equivalent between patients who had R1 and R0 resections after chemotherapy (40 vs. 55%, p = 0.104 and 9 vs. 22%, p = 0.174, respectively) [181]. However, a recent metanalysis showed that for patients having neoadjuvant therapy, an R1 margin was associated with a risk difference (RD) of 0.09 (95% CI 0.02–0.17, p = 0.01) compared to R0 margins, but there was no significant difference for patients having adjuvant chemotherapy [182].

As before, tumour biology plays an important role when considering the impact of margin status. Interestingly, Margonis et al. showed no difference in long-term outcomes between patients with R0 and R1 resection who had KRAS mutations, but for those with wtKRAS tumours, an R1 resection was associated with a worse 5-year OS (HR 2.16, 95% CI 1.42–3.30, p < 0.001) [183]. Hatta et al. similarly showed no increased risk for those with mtKRAS tumours and R1 resection but a worse 5-year OS for those with wtKRAS tumours and margins <1 mm (HR1.77, 95% CI 1.23–2.58, p = 0.002) [184]. Iwaki et al. showed that R1 resection was associated with higher local recurrence rates, irrespective of KRAS status [185]. Xu et al., on the other hand, showed no difference in the 5-year OS between R0 and R1 resections for those with wtKRAS tumours and response to neoadjuvant chemotherapy, but mtKRAS status and poor response to chemotherapy were both negative predictors for survival when coupled with an R1 resection margin [186].

The type of R1 margin may also impact oncological outcome—Vigano et al. showed that an involved parenchymal margin is associated with a poorer long-term outcome, but when that margin occurs at the point of detachment from a blood vessel (R1vasc), there is no difference in local recurrence rates when compared to an R0 resection [187].

### 7.6. Two-Stage Hepatectomy

Two-stage hepatectomy (TSH) was developed as a surgical approach to manage bilobar metastatic disease, particularly when there are concerns about insufficient FLR for a single-stage resection [188]. The approach typically centres around “clearing” the left lobe of the liver of lesions with PPS or ablation, followed by right-sided PVE and interval right hepatectomy following hypertrophy of the planned FLR [189]. Long-term survival can be achieved with TSH, with a multi-institutional study from the USA showing a 5-year OS of 44% and a DFS of 18% [190]. A 5-year DFS as low as 8.9% has been reported; however, repeat hepatectomy can be safely performed in carefully selected patients, showing that options for salvage remain even after TSH [191].

Two major limitations to TSH exist. There are significant perioperative risks, with an incidence of major postoperative complications reported in approximately 40% of patients [192]. There also remains the issue of failing to proceed to the second stage of surgery, either due to disease progression or complications from the first stage of surgery, which is seen in 18–30% of patients and is associated with poor long-term survival [193,194].

### 7.7. Associating Liver Partition and Portal Vein Ligation for Staged Hepatectomy (ALPPS)

The ALPPS procedure is an evolution of the concept of TSH, whereby an operative exploration, ligation of the right portal vein, and in situ splitting of the liver parenchyma are carried out simultaneously, achieving the desired hypertrophy of the FLR in as little as 9 days [195]. The key advantage that this approach has over TSH is that a far higher proportion of patients proceed to the second stage. Kambakamba et al. reported successfully performing the second-stage procedure in 95% of patients with a median hypertrophy of the FLR of 53.7% after six days [196]. Initial results from ALPPS have also shown, however, a significant incidence of perioperative major morbidity and a 90-day mortality rate of 9% [197]. With the adoption of more careful patient selection and more conservative resection at the first stage (“mini ALPPS”), morbidity and mortality rates have improved significantly over time, with a 90-day mortality rate of 4% reported by the ALPSS registry in 2017 [198]. Minimally invasive surgery has also been shown to improve perioperative outcomes in ALPPS [199].

The adoption of ALPPS as the procedure of choice over TSH is far from universal, and there remains considerable inter-centre variation in utilisation and perioperative outcomes [200]. Certainly, concerns exist about the risk of perioperative morbidity and mortality, while the interval between the first and second stage in TSH may also allow a test of time for unfavourable biology to declare itself [201].

The LIGRO RCT randomised 97 patients with CRLM unsuitable for one-stage hepatectomy due to FLR < 30% post-chemotherapy to either ALPPS or TSH. The trial showed that ALPPS is associated with a higher resection rate (92% vs. 57%, p < 0.0001), with no difference in major complications (43% vs. 43%, p = 0.99) and equivalent 90-day mortality (8.3% vs. 6.1%, p = 0.68) [202]. Long-term outcomes from the trial show an estimated median survival of 46 months (95% CI 34–59 months) for patients randomised to ALPPS (n = 48) and 26 months (95% CI 16–36 months) for patients randomised to TSH (n = 49) (p = 0.028) [203]. A subsequent metanalysis, incorporating the LIGRO trial and four retrospective studies, showed increased major postoperative morbidity for patients undergoing ALPPS (RR 1.46, 95% CI 1.04–2.06, I^2^ = 0%) but no difference in OS and DFS [204].

The so-called “rescue ALPPS” has a role in which PVE embolisation fails to achieve the desired hypertrophy in the FLR. In this setting, ALPPS has been shown to be effective, achieving a median increase in the FLR of 61.8% in one series [205]. The emergence of LVD, as discussed above, may, however, supplant the role of ALPPS going forward—a recent metanalysis showed no difference between LVD and ALPPS in the hypertrophy of the FLR but significantly reduced morbidity and mortality with LVD [206].

### 7.8. The Role of Minimally Invasive Surgery (MIS)

The emergence of MIS for hepatectomy has shown the potential to transform perioperative outcomes for patients, with a reduction in the LOS, lower perioperative morbidity, and quicker return to function [207]. MIS resection has also been shown to allow patients to return to systemic chemotherapy quicker than with open surgery [208].

The first RCT comparing laparoscopic liver resection (LLR) with open liver resection (OLR) incorporated 20 patients in each arm and showed shorter LOS for LLR, with no difference in perioperative morbidity [209]. The subsequent OSLO-COMET RCT was a single-centre RCT that randomised 133 patients to LLR and 147 to OLR [210]. The trial showed no difference in perioperative mortality but reduced postoperative morbidity (19 vs. 31%, p = 0.02) and shorter LOS for LLR. No difference was seen in the 5-year OS or RFS between LLR and OLR (54% vs. 55%, p = 0.67, and 30% vs. 36%, p = 0.57 respectively) [211]. The LapOpHuva RCT randomised 96 patients with CRLM to LLR and 97 to OLR [212]. They showed no difference in major postoperative morbidity, 5-year OS (49.3% vs. 47.4% p = 0.82), or 5-year DFS (22.7% vs. 23.9%, p = 0.23) but reduced LOS (4 vs. 6 days, p < 0.001) and reduced overall postoperative morbidity (11.5% vs. 23.7%, p = 0.025) for LLR. Even in the setting of redo surgery for recurrent CRLM, LLR is associated with good long-term survival for patients with resectable disease [213].

More recently, robotic liver resection (RLR) has emerged as an option for patients with CRLM, with reduced perioperative morbidity when compared to OLR [214]. Chang et al. conducted a single-centre RCT including 171 patients scheduled for simultaneous rectal and liver resection for metastatic rectal cancer in a 1:1 fashion to robotic or open surgery [215]. Patients in the robotic surgery arm (n = 86) experienced fewer postoperative complications (31.4% vs. 57.6% p = 0.014) and shorter LOS (8.0 days vs. 10.7 days, p < 0.001) than the open surgery arm (n = 85), with no difference in 3-year OS and DFS. It should be noted, however, that only 20 patients (23.2%) in the robotic group and 18 patients (21.1%) in the open group received neoadjuvant treatment of any form, and that rates of adjuvant treatment are not reported, potentially limiting the generalisability of their results. A metanalysis by Wong et al. showed that RLR is associated with fewer major complications (RR 0.45, 95% CI 0.22–0.94, p = 0.03) and shorter LOS (MD −2.57 days, 95% CI −3.31–−1.82, p < 0.001), but longer operative times (MD + 61.47 min, 95% CI 7.03–115.91, p = 0.03) than OLR [216]. Concern about the higher perioperative costs of RLR is sometimes cited as a limiting factor of a broader adaptation of the robotic approach, but multiple cost analyses have shown that this is offset by lower postoperative costs, and so RLR has lower overall direct hospital costs than OLR [217,218].

RLR has potential advantages over LLR in that it provides improved stability and flexibility than laparoscopy [219]. When compared to LLR, RLR has been shown to have fewer major postoperative morbidities (OR 0.6, 95% CI 0.4–0.9, p = 0.01) and shorter LOS (MD −0.64, 95% CI −0.78–−0.49, p < 0.001) [220]. Chong et al. showed that robotic right hepatectomy/extended right hepatectomy was associated with fewer conversions to open surgery and shorter postoperative LOS when compared to LLR [221]. Cipriani et al. showed that for more complex resections, RLR had superior perioperative outcomes in comparison to LLR, but there was no advantage to the robotic approach for moderate- to low-complexity hepatectomy [222]. Interestingly, the robotic approach appears to be most advantageous for lesions located posterosuperiorly within the liver, which are more technically challenging laparoscopically [223]. Data from the International Consortium on Minimally Invasive Liver Surgery (I-MILS) shows that patients undergoing RLR achieved textbook outcomes more frequently than those undergoing LLR (78.3% vs. 71.8%, p < 0.001), although the difference in a subgroup analysis of major and posterosuperior segments (I, IVa, VII, VIII) was not significant [224].

### 7.9. Ablation as an Adjunct to Surgery

Tumour ablation—by microwave ablation (MWA) or radiofrequency ablation (RFA)—can effectively treat CRLM up to 3 cm in diametre, particularly when resection is not feasible but concerns remain about long-term oncological outcomes [225]. The recently published prospective cohort MAVERRIC study included patients with resectable CRLM and ≤5 lesions, all < 3 cm, who underwent MWA, with a comparison to a matched historic cohort who underwent liver resection [226]. Patients treated with ablation had fewer post-procedural complications (10% vs. 30%, p < 0.01), and an identical 3-year OS when compared to the historic liver resection cohort. COLLISION is an ongoing RCT comparing surgery and microwave ablation for patients with at least one CRLM under 3 cm and may provide evidence to support avoiding surgery for smaller, ablatable tumours [227]. A combined approach with simultaneous ablation and resection for a high burden of CRLM has also been shown to be safe and effective, with a 5-year OS of 33.2% and a DFS of 23.5% [228]. Improvements in ablation technology may add strings to the surgeon’s bow in addressing patients with complex, bilobar disease and emphasise the benefits of a multidisciplinary approach to CRLM.

### 7.10. Liver Transplantation for CRLM

Historically poor long-term survival did not justify liver transplantation (LT) for unresectable CRLM, but recent evidence is challenging that paradigm [229]. To date, the uptake remains low, with Sasaki et al. reporting that 46 patients underwent LT for CRLM in the United States between 2017 and 2022 [230].

The SECA-I study was a Norwegian prospective pilot study of 21 patients who underwent LT for unresectable CRLM without extrahepatic disease [231]. Although the median DFS was 10 months, long-term follow-up showed a 5- and 10-year OS of 43.5% and 26.1%, respectively [232]. Based on the outcome of the SECA-I study, the authors proposed the Oslo prognostic score, consisting of a tumour size > 5.5 cm, less than two years from primary colon resection to LT, progressive disease at the time of LT, and preoperative CEA >80 µg/L as poor prognostic features.

The subsequent SECA-II prospective trial of 15 patients with considerably stricter inclusion criteria and a median Oslo score of 1 showed that LT for unresectable CRLM had a 5-year OS of 83% [233].

The results of the SECA trials show that careful patient selection in the context of understanding tumour biology is key. A full discussion of the role of LT in CRLM is, however, beyond the scope of this review.

## 8. Conclusions

**Figure 1:**
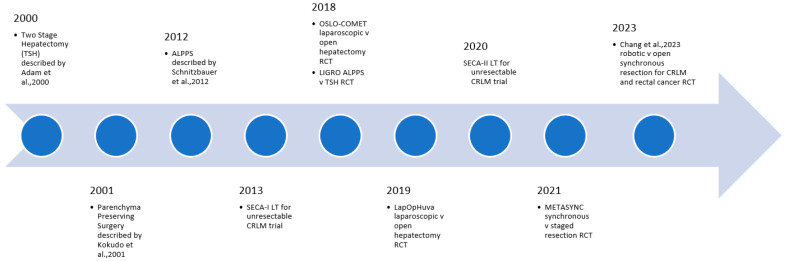
Selected key recent developments in surgical technique for CRLM [173,188,195,215].

**Figure 2:**
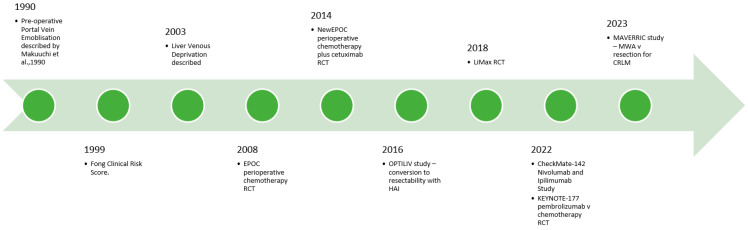
Selected key recent developments in surgical adjuncts and local and systemic options for CRLM [149].

Precision surgery provides enormous potential benefits to patients with CRLM. As our understanding of tumour biology improves, careful patient selection is crucial to ensuring good perioperative and long-term outcomes. Immunotherapy represents a particularly exciting development in the management of CRLM, but the surgeon must ensure adequate volume and quality of the liver prior to embarking on resection. In the operating theatre, the advent of parenchyma-preserving, minimally invasive approaches to liver resection allows for a reduction in the surgical insult to the patient, without compromising patient outcomes. As the incidence of CRLM continues to rise globally, the challenge for the surgeon is to assimilate the ever-expanding data on the management of CRLM and tailor it to each specific patient and scenario (Figure 1 and Figure 2).
